# The Epistemic Revolution Induced by Microbiome Studies: An Interdisciplinary View

**DOI:** 10.3390/biology10070651

**Published:** 2021-07-12

**Authors:** Eric Bapteste, Philippe Gérard, Catherine Larose, Manuel Blouin, Fabrice Not, Liliane Campos, Géraldine Aïdan, M. André Selosse, M. Sarah Adénis, Frédéric Bouchard, Sébastien Dutreuil, Eduardo Corel, Chloé Vigliotti, Philippe Huneman, F. Joseph Lapointe, Philippe Lopez

**Affiliations:** 1 Institut de Systématique, Evolution, Biodiversité (ISYEB), Sorbonne Université, CNRS, Museum National d’Histoire Naturelle, EPHE, Université des Antilles, 75005 Paris, France; marc-andre.selosse@mnhn.fr (M.A.S.); eduardo.corel@upmc.fr (E.C.); philippe.lopez@upmc.fr (P.L.); 2 Micalis Institute, INRAE, AgroParisTech, Université Paris-Saclay, 78350 Jouy-en-Josas, France; philippe.gerard@inrae.fr; 3 Environmental Microbial Genomics, Laboratoire Ampère, École Centrale de Lyon, CNRS, University of Lyon, 69134 Ecully, France; catherine.larose@ec-lyon.fr; 4 Département Agronomie Agroéquipements Elevage Environnement, UMR 1347 Agroécologie (INRA/AgroSup/Université de Bourgogne), 26 Bd Docteur Petitjean, BP 87999, CEDEX, 21079 Dijon, France; manuel.blouin@agrosupdijon.fr; 5 Sorbonne Université, CNRS, AD2M-UMR7144, Station Biologique de Roscoff, 29680 Roscoff, France; not@sb-roscoff.fr; 6 PRISMES—Langues, Textes, Arts et Cultures du Monde Anglophone—EA 4398, Université Sorbonne Nouvelle and Institut Universitaire de France, 75005 Paris, France; lilianecampos1@gmail.com; 7 CERSA, UMR 7106 (CNRS—Université Paris II Panthéon-Assas), 10 rue Thénard, 75005 Paris, France; geraldine.Aidan@cnrs.fr; 8 Faculty of Biology, University of Gdańsk, ul. Wita Stwosza 59, 80-308 Gdańsk, Poland; 9 PILI, 16 Avenue du Bas Meudon, 92130 Issy-les-Moulineaux, France; mariesarah.adenis@gmail.com; 10 Département de Philosophie, Université de Montréal, Montréal, QC H3C 3J7, Canada; f.bouchard@umontreal.ca; 11 Centre Gilles Gaston Granger, CNRS, UMR7304 Université d’Aix-Marseille—Site Schuman, Maison de la Recherche, 29, Avenue Robert Schuman, 13621 Aix-en-Provence, France; seb.dutreuil@gmail.com; 12 UMR MIA-PARIS, 16 Rue Claude Bernard, 75005 Paris, France; chloe.vigliotti@agroparistech.fr; 13 Institut d’Histoire et de Philosophie des Sciences et des Techniques, CNRS/Université Paris I Sorbonne, 13 rue du Four, 75006 Paris, France; philippe.huneman@gmail.com; 14 Département de Sciences Biologiques, Complexe des Sciences, Université de Montréal, 1375 Avenue Thérèse-Lavoie-Roux, Montréal, QC H2V 0B3, Canada; francois-joseph.lapointe@umontreal.ca

**Keywords:** microbiomes, evolutionary microbiology, microbial ecology, networks, individuals, selection, philosophy of biology, humanities, visual art, literature, law

## Abstract

**Simple Summary:**

This interdisciplinary study, conducted by experts in evolutionary biology, ecology, ecosystem studies, arts, medicine, forensic analyses, agriculture, law, and philosophy of science describe how microbiome studies are convergently affecting the concepts and practices of diverse fields and practices, that now consider microbiomes within their legitimate scope. Consequently, it describes what seems to be an ongoing pluridisciplinary epistemic revolution, with the potential to fundamentally change how we understand the world through an ecologization of pre-existing concepts, a greater focus on interactions, the use of multi-scalar interaction networks as explanatory frameworks, the reconceptualization of the usual definitions of individuals, and a de-anthropocentrification of our perception of phenomena.

**Abstract:**

Many separate fields and practices nowadays consider microbes as part of their legitimate focus. Therefore, microbiome studies may act as unexpected unifying forces across very different disciplines. Here, we summarize how microbiomes appear as novel major biological players, offer new artistic frontiers, new uses from medicine to laws, and inspire novel ontologies. We identify several convergent emerging themes across ecosystem studies, microbial and evolutionary ecology, arts, medicine, forensic analyses, law and philosophy of science, as well as some outstanding issues raised by microbiome studies across these disciplines and practices. An ‘epistemic revolution induced by microbiome studies’ seems to be ongoing, characterized by four features: (i) an ecologization of pre-existing concepts within disciplines, (ii) a growing interest in systemic analyses of the investigated or represented phenomena and a greater focus on interactions as their root causes, (iii) the intent to use openly multi-scalar interaction networks as an explanatory framework to investigate phenomena to acknowledge the causal effects of microbiomes, (iv) a reconceptualization of the usual definitions of which individuals are worth considering as an explanans or as an explanandum by a given field, which result in a fifth strong trend, namely (v) a de-anthropocentrification of our perception of the world.

## 1. Introduction

It is uncontroversial that microbiome studies are starting to significantly transform our understanding of the functional, eco-systemic, and evolutionary importance of microbial communities. Many novel scientific concepts have resulted from recent microbiome research, e.g., the holobiont/meta-organism concept, co-evolution, microbiome functions and management, to name a few [1,2,3,4,5,6]. However, what is less appreciated is that because active microbes are (almost) ubiquitous, a growing number of fields and practices, originally traditionally centered on very distinct objects and questions, nowadays consider microbes as part of their legitimate focus. Beyond ecosystem studies, microbial, and evolutionary ecology, this growing interest for microbiome studies can also be found in arts, in medicine, in agriculture, in law, and in philosophy of sciences. All these fields and their associated practices are in fact undergoing some conceptual rethinking that is emerging from the still striking realization that most species, ours included, do not live alone, but are always surrounded and shaped by microbiomes. Therefore, below we review some of the reasons why microbiome studies may act as unexpected unifying forces across very different disciplines and argue that a global epistemic revolution induced by microbiome studies is possibly ongoing.

## 2. A Cross-Disciplinary Perspective

On 23 March 2021, an interdisciplinary conference, organized by E. Bapteste, E. Corel, P. Lopez, and C. Vigliotti, entitled “New Challenges Induced by Microbiomes”, was held virtually in Paris. This event featured fourteen renowned experts from fields as diverse as law, microbial ecology, microbial evolution, visual arts, forensic sciences, philosophy of biology, popular scientific writing, and comparative literature. This meeting emerged from the recognition that microbiome studies, i.e., the studies of communities of microbes in interaction with other microbial, animal, or plant hosts or with their environments, fueled by the remarkable progress made in environmental genomics, were starting to significantly transform our understanding of the functional, eco-systemic, and evolutionary importance of microbial communities, and because we agreed with [5] that microbiome studies should be cross-disciplinary.

Invisible for a long time, then, once discovered, commonly seen as potential enemies of mankind, microbes (protists, archaea, bacteria, and viruses), the oldest, most abundant life forms on Earth, are in the process of conquering the valorizing titles of critical biological, ecological, and evolutionary players. From our human perspective, microbes and microbiomes are increasingly perceived as important entities in the past, present, and future developments of our own societies. Because active microbes are (almost) everywhere, in us, on us and around us, many seemingly separate academic fields outside microbiology, although traditionally centered on very distinct objects and questions, nevertheless share a common interest. Namely, a growing number of fields and practices nowadays consider microbes as part of their legitimate focus. Therefore, beyond microbiology, microbiome studies are now inspiring new knowledge and practices in multiple research and artistic fields. The fact that microbiomes feature as objects of study in multiple fields suggests that microbiome studies may in turn act as unexpected unifying forces across very different disciplines and that they could contribute to a possible global ‘epistemic revolution induced by microbiome studies’, simultaneously (and as it seems deeply) transforming our knowledge and practices far beyond their original field of discovery. Precisely, this conference investigated whether research on microbiomes were starting to induce convergent changes across distinct disciplinary fields, and, if such convergences occurred, to sketch a first, big picture of the general, ongoing conceptual rethinking that emerges from the still striking realization that most species, including ours, do not live alone, but are always surrounded and shaped by microbiomes.

The talks, followed by live questions with a connected audience, were divided into four main sessions, namely “Microbiomes, as novel major biological players”, “Microbiomes, as new artistic frontiers”, “New applied uses of microbiomes”, and “Microbiomes, as root causes for novel ontologies”.

It is increasingly appreciated that microbes and viruses, in particular the ones living in the oceans, drive biogeochemical cycling on a global scale [7]. It remains to be characterized however how this scientific realization occurred and what causal processes and biological mechanisms confer the role of major ecological and evolutionary players to microbial communities.

Dr. Sébastien Dutreuil (CNRS, Centre Gilles Gaston Granger, Aix-Marseille University, France), a Historian and Philosopher of the Environment, addressed the issue of the recognition of the role of microorganisms in the Earth sciences from a historical perspective. He opened the first session by providing such a perspective on the studies by James Lovelock and Lynn Margulis on bacteria, Gaia and the global environment in the 1960s and 1970s. Gaia was presented as the name of a hypothesis, namely the idea that life may regulate the global environment (atmospheric and oceanic composition as well as the climate); and as the name of a new entity, composed by the total ensemble of living beings and the environment with which they interact. Dr. Dutreuil stressed how Lovelock and Margulis’ interdisciplinary collaboration, between a chemist and a microbiologist, was critical for the recognition of bacteria’s influence on Earth’s biogeochemistry and climate, and also how Lovelock’s personal, scientific, and political thoughts on global pollution contributed to bring forward the recognition of a bacterial impact at the scale of the planet [8,9] (Table 1). Dr. Dutreuil concluded that in the Earth sciences, the importance of bacteria’s influence on the global environment has been put forward in the wake of the Gaia hypothesis and of the rise of biogeochemistry in the 1970s and 1980s.

Next, Dr. Fabrice Not (CNRS, Adaptation and Diversity in Marine Environment, Sorbonne-University, France), an expert in microbial oceanography, tackled the issue of why a planetary-scale understanding of the ocean ecosystem, particularly in light of climate change, although desirable, remained a most challenging scientific goal, hindered by well-characterized limits regarding our understanding of the composition, functioning, and evolution of natural microbial communities [10]. Dr. Not illustrated this claim through an analysis of the most recent research on marine microbiomes, making it clear that, fifty years after the work initiated by Lovelock, our understanding of the marine microbiomes had now turned into a major scientific issue, and that, without explicit consideration of these microbiomes, major biogeochemical cycles impacting the planet, in particular the carbon cycle, could not be properly modeled. Yet, Dr. Not also showed that, while microbial studies were starting to unravel the role of microbes in the functioning of oceanic ecosystems, the functions and interactions of a shockingly large proportion of marine microbial genes and species remained problematically unknown [10,11,12,13]. Finally, Dr. Catherine Larose (CNRSEEA/Ampère, Lyon, France), a microbial ecologist, closed the first session by reflecting on the diversity and the evolution of microbial communities in Polar Regions. She elaborated her work within the current general consensual context that ‘we must learn not just how microorganisms affect climate change but also how they will be affected by climate change and other human activities’ [6]. Dr. Larose explained that, while Polar Regions are intrinsically involved in global cooling through a number of feedback mechanisms, these regions are also transforming due to human actions, with huge consequences on the microbial ecology of the cryosphere. Critically, these ecosystems are largely inhabited by microbial cells, that evolved over various distinct time periods, and that may switch between active and dormant lifestyles when triggered by the prevailing environmental conditions [14,15]. Therefore, Dr. Larose concluded that it is difficult to predict and manage the dynamics and functions of microorganisms in cold habitats, since much of the current research is dominated by empirical approaches [16]. To reach the consensual goal mentioned above, the use of ecological modeling and the development of new predictive models that integrate novel approaches to conceptualize communities and their functions appears necessary to help overcome these challenges.

**Figure 1:**
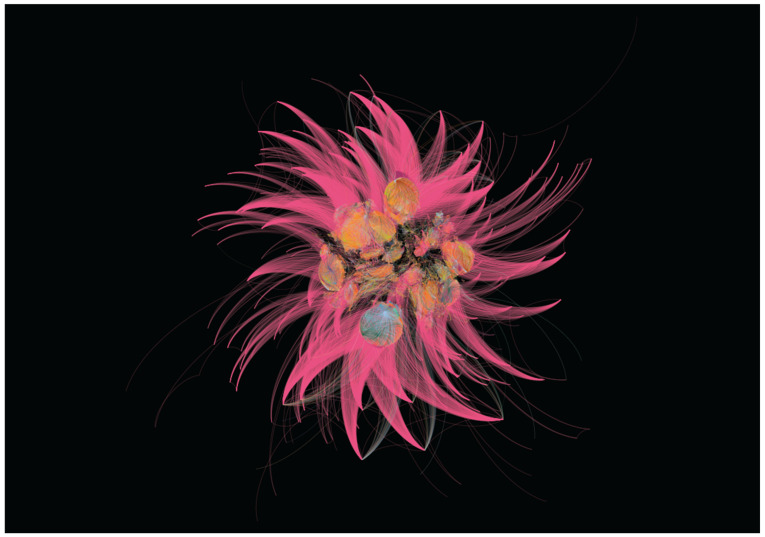
Six hundred and fifty handshakes. This piece of art by the bioartist François-Joseph Lapointe belongs to the series ‘1000 handshakes’. For this project, the artist shook hands with as many people as possible in various cities, gradually changing the microbial community in the palm of his right hand. Periodically, assistants have taken a skin microbiome sample and the DNA collected has been sequenced and analyzed to generate sequence similarity networks. Two bacteria (nodes) are connected in the network when their genetic sequences are more similar than a fixed threshold (90%), with different clusters corresponding to distinct bacterial families and different colors representing the microbiome samples collected at regular intervals (i.e., every 50th handshakes).

The second session was inaugurated by Dr. Liliane Campos (Junior Research Fellow of the Institut Universitaire de France and Lecturer at the Sorbonne Nouvelle, France), who analyzed the microbiome imaginary recently constructed by popular biology, drawing on a selection of texts intended for a general readership, including books for children [17,18,19,20,21,22,23,24]. Dr. Campos presented their recurrent metaphors, the affects these images suggest, and the epistemological shifts they perform. She concluded that a transition had been set in motion: metaphors involving microbes are evolving from military to environmental images, such as the zoo or the garden, and stories told about microbes tend to displace agency away from human individuals, towards collective actors. Next, Dr. Marie-Sarah Adénis (Creative Director at PILI, Member of the artistic and scientific advisory board ‘La Chaire arts and sciences’, France), demonstrated the unique potential of visual arts to contribute to radically updating popular images of microbes. This case is for example illustrated by Pr. Lapointe’s artistic practice, which revolves around microbes and the dynamics of contamination. By engaging in a variety of experimental projects, Pr. Lapointe collects microbiome data to track changes in his bacterial identity. Through physical engagement and audience participation, his work raises awareness about contagion at the social, individual and microbial levels, thus exhibiting how our interactions with others shape the microbes between us, and how it changes over time to reveal who we are (Figure 1). Dr. Adénis concluded that visual arts were needed to establish the complexity and fundamental ecological roles of microbes, as this artistic medium effectively complements scientific discourse. This view was also supported during the next talk. A successful scientific writer of both popular sciences essays and of a graphic novel on microbiomes [25,26,27], soil microbiologist Pr. Marc-André Selosse (Muséum national d’Histoire naturelle, ISYEB UMR 7205, Paris, France), explained that owing to their minute sizes, microbes remained invisible to the public, with the noticeable exception of some of their effects, first on our species and second on our environment. In addition, Pr. Selosse stressed that our strongly entrenched anthropo-zoo-centrism constituted a further major challenge on the road towards a genuinely broad diffusion of the scientific concepts necessary to accurately describe microbial biology and the striking yet overlooked diversity of microbiomes. In his eyes, we are living in a microbial world and we need to understand non-animal organisms as they are in themselves. If we consider only their direct links to us, or overuse metaphors derived from animals (such as intelligence, sensitivity, etc.), we turn these organisms into pale copies of our own essence. Two scientific editors of the major French popular science magazine ‘Pour la Science’ (Marie-Neige Cordonnier and Loïc Mangin) confirmed a prevalent popular interest for research on microbiomes and human health over other popular scientific topics and anticipated a growing interest for the popularization of scientific analyses of non-human microbiomes and of the fundamental, philosophical consequences of microbiome studies.

Consistent with this trajectory, progressively moving away from purely medical interests, Dr. Philippe Gérard (Micalis Institute, INRAE, AgroParisTech, Paris-Saclay University, France) started the third session on the novel uses of microbiomes by a talk on microbiomes and health. In the last decade, the relationship between the gut microbiome and health and disease has been extensively documented. However, most studies are limited to associations, and assessing causality remains a challenge. Gut microbiota transplants in animal model have therefore become a gold standard to confirm the causative role played by the microbiome in a defined pathology. In this context, Dr. Gérard recalled that while studies of microbiomes were already carried out by Pasteur in 1885, and effective germfree models exists since the 1940s, it has only recently become widely appreciated that microbial dysbioses are causally involved in a surprisingly large array of diseases (Table 1), to the point that the traditional experimental protocol dating back to Koch’s work, which demonstrates the causal implication of a single microbial species in a pathology and is known as the Koch postulate, has now been expanded to routinely assess the causality of entire microbial communities [28] (see below). As examples, he presented how human microbiota transplants to germfree mice allowed researchers to demonstrate causality, linking the microbiome to alcoholic liver disease [29] and to hypercholesterolemia [30]. Dr. Gérard stressed that, as microbial communities causally impact their hosts, using microbiomes for medical transplants alters individual phenotypes, which presents us with novel ethical issues. Next, Pr. François-Joseph Lapointe (Faculté des arts et des sciences—Département de sciences biologiques, Montréal, Canada) introduced the promises and challenges of the development of forensic methods based on the analyses of post-mortem microbiome dynamics, and described the imminent use of microbiomes as signatures on a crime-scene [31,32,33,34,35,36,37,38]. Pr. Lapointe proposed that statistical analyses of microbiomes would soon be used as effective proxies of their host identities, across genders and across species, contributing to more acute reconstitutions of crimes and crime scenes, and also, through their changes over time after their host’s death, providing a novel microbial clock, ticking throughout the various stages of host corpse decomposition with greater accuracy than insect-based analyses. Finally, an expert in law and legal theory, Dr. Géraldine Aïdan (UMR 7106, Université Paris II Panthéon-Assas, France) explained how political considerations could be invoked to grant microbiomes the status of new right-holders. Dr. Aïdan confirmed that, like some glaciers or rivers and several other non-human entities (Table 2) that benefit from this legal status, nothing opposed this possibility for microbiomes. The condition for this is that microbiomes, like any other non-human entities presently considered to be right-holders, could be represented in courts by a human. However, the main problem in current law specifically concerns the legal qualification of the microbiome, in particular in health law (i.e., should microbiomes be considered as “elements and products resulting from the human body”, “Medicine”, or should they receive some sui generis qualification? [39,40]). Moreover, new questions are emerging around the microbiota as a legal actor. Accordingly, Dr. Aïdan stated that it is timely to determine the best way to ensure the legal protection of a microbiome, when this microbiome is recognized as a fundamental element of the human ecosystem (both physically and psychologically) or of the general ecosystem of the planet. She envisioned two legal strategies to reach this goal: first, entities associated with microbiomes could benefit from the status of legal subject; second, microbiomes themselves could benefit from such status. Dr. Aïdan noted that the current political context, by promoting an evolution towards an ecocentric conception of law, in which species and ecosystems are morally significant, and the end of a form of anthropocentrism, certainly encourages the second way. However, she also questioned the use and feasibility of this legal technique, arguing that the prohibition of certain human behaviors against microbiota may be sufficiently efficient. In any case, Dr. Aïdan stressed that prior redefinition of our human identity, in that it is partly shaped by our microbiomes, was necessary to grant rights to human-associated microbiomes or to a collective of human hosts and microbes with emergent properties, especially if some form of interiority resulted from these interactions [41]. Furthermore, Dr. Aïdan noticed that due to the ecological connections between various microbiomes and various hosts, granting rights to microbiomes would instate a web of right-holders, contrasting with the common idea that a hierarchical scale allows a simpler ranking of right-holders based on their importance and phylogenetic proximity to humans.

**Figure 2:**
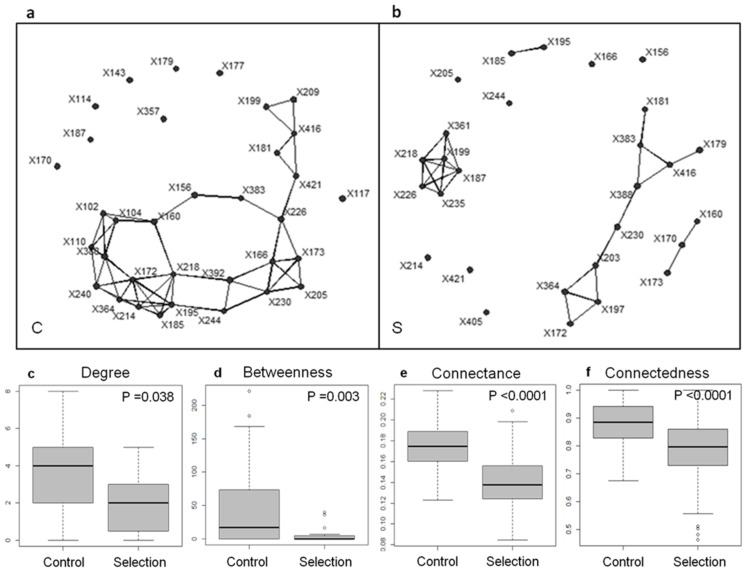
An example of a network-based analysis of microbiome selection (from [40]). The co-occurrence matrices of the T-RFLP-defined genetic units present after 21 generations were used to build interaction networks for (**a**) the control (C) and (**b**) the selection treatment for low CO_2_ emission (S). When two dots are connected by lines, it means that the abundances of the genetic units were significantly correlated (Spearman correlation coefficient; N = 6; *p* < 0.05). The interaction networks were used to calculate several network indices (**c**–**f**) as follows. The average degree (**c**) is the average number of interactions engaged in by one genetic unit (equal to 0 for an unconnected unit), providing an estimate of network complexity. The average betweenness (**d**) is the average number of shorter chains going through one node, which can signal the presence of keystone species in the network. The connectance (**e**) is the proportion of possible links between species that are actually realized. The connectedness (**f**) is the probability that at least one chain exists between any pair of units, which quantifies all the direct and indirect interactions within the network. The networks were bootstrapped (200 random samples from each group’s pool of genetic units) to determine (**e**) average connectance and (**f**) average connectedness. The values of these indices were compared for the control and selection treatment using Wilcoxon rank-sum tests (employing a continuity correction for non-parametric distributions).

The final session dived further into the philosophical issues raised by microbiomes. These questions are increasingly debated [42,43,44,45], with a major emphasis on the need to redefine or adjust the concept of identity and the concept of unit of selection to account for microbiomes. First, Pr. Manuel Blouin (AgroSup Dijon, UMR 1347 Agroécologie, INRA, Université de Bourgogne, UBFC, France) produced a strong case for the ability to artificially select microbiomes (Figure 2). To support or invalidate the idea that ecosystems are units of selection, he indicated that one should ideally observe natural selection of macroscopic ecosystems in the wild. Due to methodological and time constraints, Pr. Blouin proposed to artificially select microbiomes in the laboratory and to assess the relevance of considering ecosystems as units of selection based on these experimental results, as Darwin did with the artificial selection of pigeons to support the theory of evolution by natural selection [46]. He presented an experiment that consists of (i) growing a given number of microbial communities, considered as a lineage, in the wells of a micro-plate for a given “generation time” (or growth cycle); (ii) selecting some of the microbiomes among those of a lineage based on the value of a target property (e.g., degradation of a pollutant, production of biomass, emission of CO_2_…); (iii) inoculating a new generation of microbial communities in wells with a new nutritive solution; and (iv) reiterating the procedure for several “generations”, as is done, by analogy, to breed crops or dogs [47]. In line with seminal work on simple two-species communities of beetles [48,49] and with pioneering studies on far more complex microbial communities [50,51], Pr. Blouin concluded that despite being multi-specific, phenotypic properties of microbial ecosystems can be changed in the expected direction (here low CO_2_ emission), as compared with a control where microbial communities are randomly chosen. Since changes in CO_2_ emission were associated with differences in indices describing the interaction network of the microbial community (Figure 2), Pr. Blouin assumes that evolutionary changes occurred at the level of the community structure, rather than at the level of the genome of a specific species, in line with modeling results [52,53]. According to him, microbiomes thus deserved to be considered as bona fide units of selection, hence as novel evolutionary individuals in their own right. Significantly, their artificial selection could be of great interest in applied realms such as environment, agriculture, or medicine.

Moving beyond the approach of artificial selection to test for the existence of underappreciated units of selection, Ford Doolittle (Dalhousie University, Canada), a famous evolutionary biologist and now recognized philosopher of biology, introduced another theoretical type of selection, which he called ‘clade selection’. A clade includes all the descendants of a single ancestor. Thus, by definition, clades can only persist or go extinct. Moreover, any competition between clades must then be for persistence: it cannot be for reproduction, because any descendant of a clade will still belong to that clade. Simply put, a clade X can be said to be fitter than a clade Y if X has a higher propensity to persist for Z amount of time than Y. Precisely, Doolittle argued that clades are less likely to go extinct, when the species that made them up are more numerous, ecologically diverse, geographically dispersed, and possibly cooperate with one another within the clade (e.g., by lateral transfers of metabolites or genes). Remarkably, all these features proposed to enhance the persistence of a clade are not properties carried by any single species. Consequently, features supporting clade persistence cannot be selected at the species level. Instead, Doolittle proposed that some selection can act at the level of clades, through differential persistence through time. He also argued that, whereas the intuitive idea that an entity as complex and phylogenetically diverse as Gaia could not evolve by natural selection, clade selection could in fact support the Gaia hypothesis in a strong form, when selective pressures favor the persistence of clades whose species richness, diversity, and dispersal are enhanced by interactions between those species and by interactions between those species and their environment, which result in global homeostatic mechanisms. Assuming that clade selection could be acting within interacting communities and environments and be responsible for the evolutionary persistence of as large an eco-system as Gaïa [54,55,56], this makes it possible to reintegrate complex entities, including Gaia, within the Darwinian framework. Consistently, Pr. Doolittle’s work hinted at the possibility that specific microbiomes, too, could be affected by clade selection, and be the target of a selection for persistence, which would allow ‘Darwinizing microbiomes’, i.e., to integrate, despite their compositional diversity, some microbiomes amongst the list of genuine selective units that may evolve on the planet, when they can be explained by selection on persistence.

Next, the philosopher of biology, Pr. Frédéric Bouchard (Faculté des arts et des sciences, Université de Montréal, Canada), reinforced that claim by illustrating how microbiome studies were offering a decisive support to the philosophical concept of transient biological individuality [57,58,59,60]. Standard accounts of evolutionary success focus on populations of homogeneous biological individuals that have fixed boundaries and that reproduce. For this reason, standard accounts focus on fitness in terms of differential reproductive success (or more abstractly in terms of replication). As microbiome research highlights, evolution and nature are often much more complex. In community and ecosystem ecology and in microbiome research in particular, we have what seem to be adaptations of multi-species assemblages. For these complex systems and for evolutionary systems in general, Bouchard [61,62] argued that maximization of relative reproductive success is only one very common strategy used by lineages to increase evolutionary success, but that the property that is actually being maximized across all biological systems is increased Persistence Through Time. Many lineages increase their potential to persist via increased reproductive success, but many biological systems (e.g., many clonal species, communities, and ecosystems) increase their potential for persistence as response to selective pressures without or while reducing the reproductive success of their parts. This account is especially helpful for multi-species associations where unified reproductive success may well be absent, but where selection among their heterogeneous parts, and the accumulation of these changes, may lead to adaptive change that can increase the potential to persist of the whole. In this sense, there is selection for increased persistence across all levels of biological organizations.

More specifically, Pr. Bouchard explained how the interactions between hosts and microbiomes, for example in the case of *Vibrio fisherii* communities and individual bobtail squids, resulting in transiently glowing squids, could give rise to selectable traits that only existed when interactions involving microbiomes and their animal host were realized, and therefore that heterospecific evolutionary individuals, although they surely remained non-paradigmatic, were likely underappreciated and critical bona fide units of selection [58,60], detailing philosophical aspects underlying the popular studies of holobionts [3]. Finally, philosopher of science Dr. Philippe Huneman (Institut d’Histoire et de Philosophie des Sciences et des Techniques, CNRS/Université Paris I Sorbonne, France) summarized the range of changes induced by microbiome studies in evolutionary biology. Dr. Huneman noted that microbiome studies had simultaneously (i) led to novel units of selection being proposed, (ii) enhanced systemic perspectives on the concepts of identity and individuality, and (iii) encouraged researchers to complement traditional tree-based approaches to evolution, such as the use of phylogenies to describe the divergent evolution of monophyletic groups, by more general network-based frameworks, better suited to describe the evolution of microbiomes and of interacting lineages [63].

## 3. Commonalities across Novel Avenues of Research

This series of talks and the subsequent lively discussions that each of them produced allowed us to sketch a first big picture of a general, ongoing, cross-disciplinary epistemic transformation induced by microbiome studies. Indeed, several convergent emerging themes are obvious across the fields and practices that now consider microbiomes within their scope.

The first common feature of the ‘epistemic revolution induced by microbiome studies’ is an integration of concepts from the ecology theory [2] into various fields, as part of an ecologization of pre-existing concepts within disciplines, namely the recognition that communities of interacting microbes, rather than single species, evolving independently, are playing a causal role in explaining some phenomena of interest, as was recently noted by [4]. For example, the ecologization of medicine is nicely illustrated by the assumed adaptation of the original Koch postulate into a broader ecological Koch postulate. The former concept was used as a recipe to identify which given species are causally involved in a given pathology. In brief, a given microorganism is held responsible for a disease when this microorganism is obligately present in all diseased hosts, and the re-inoculation of this microorganism into a naïve host, following its isolation from the diseased hosts and growth in pure culture, results in the same diseases as in the original host. Finally, the suspected microorganism must then also be recovered from the newly diseased hosts. By contrast, in the ecological Koch postulate, the same logic is extended from single species to communities. A microbiome can thus now be held as causally involved in a disease when a dysbiotic microbiome is found in similar composition/with similar characteristics in all infected individuals, when this dysbiotic microbiome can be retrieved from the affected hosts, and when the gavage of germ-free hosts with this retrieved microbiome leads, in combination with a similar environment, to similar symptoms as in the affected individuals [28]. Similarly, in oceanography, structured microbial communities and their dynamics are explicitly coming out of what was a former black box, as they are now specifically considered as prime causal agents in the completion of biogeochemical cycles. Likewise, in agriculture, specific microbial strains (e.g., mycorrhiza or Plant Growth Promoting Bacteria) were inoculated to improve plant growth and immunity. A new trend is to inoculate entire microbiomes to reach the same goals [64].

Strikingly, while ecologists in the 20th century struggled with the idea that ecosystems are (like) organisms, be they considered as individuals endowed with metabolism [65] or as targets of group selection ensuring the cohesion of parts as individual selection does for organisms [66], recent approaches consider organisms as ecosystems [67,68]. Microbiomes are a major focus of this paradigm shift (e.g., [69]). This ecologization of traditional explanations and practices is also noticeable in the humanities. Comparative literature analyses of text involving microbiomes highlight the emergence of an eco-narration [70,71] with metaphors taken from macro-ecology to describe what looked like simple organisms before microbiome studies gained in importance. Likewise, the attribution of the status of right-holders to eco-systems, as opposed to humans or single species, develops a symbolic eco-centrism, which contributes to ecologizing the category of right-holders (Table 2).

Consistently, the second common emerging theme induced by microbiome studies in multiple disciplines is a growing interest for systemic analyses of the investigated or represented phenomena, and a greater focus on interactions as their root causes. Thus, some prominent philosophers of biology propose to give a greater evolutionary significance to transient individuals [72], which emerge from interactions with microbes, or even to consider that interactions in themselves, as opposed to individuals, in particular microbial interactions, constitute major evolutionary events, which provide a necessary scaffold for the evolution of Life on Earth, which precisely builds upon such interactions [63], despite the fact that interactions are not conventional evolutionary players. Likewise, analyses of the changes in community structures during a process of artificial selection, hence the study of the modifications before and after a selective treatment, now include not only the changes of the microbial components, but also the variations of the microbial interactions within a selected eco-system [73]. Since they emerge at the community level, changes in interactions are thus now considered as novel, essential evidence to assess the occurrence of eco-system selection.

Accordingly, microbiome studies support a third general feature that emerges in common within several disciplines: the desire to use openly multi-scalar interaction networks as an explanatory framework to investigate phenomena. The multi-scalar nature of these networks stems from the fact that microbes in interactions with their hosts and/or with their environments have typically different spatial sizes, and usually different lifespans, generation times, and turn-over rates, hence different evolutionary rates than their biological hosts, or than the physicochemical cycles to which these microbes contribute [74,75]. Thus, convergent clues that modeling multi-scalar interdependence is becoming a common goal for many disciplines acknowledging the causal effects of microbiomes can be found in the novel evolutionary models of Gaïa, in the descriptions of oceanographic models of biogeochemical cycles dynamics, or in the ecological models of microbial cryosphere dynamics that explicitly model the impact of microbiomes in a context of global warming, as well as in the emerging idea that right-holders with transient microbial components deserve to be treated as entities connected through multi-scalar interaction networks.

The fourth common feature induced by microbiome studies is a reconceptualization of the usual definitions of individuals worth considering as an explanans or as an explanandum by a given field. Thus, microbiome signatures are proposed to offer a novel proxy for their hosts identity, suggesting that, at least in a medico-legal context, and despite considerable philosophical debates on what defines individuals and identity over time in the first place [72,76,77], some practitioners are now tempted to equate (hence to reduce) the legal individuals to parts of their (changing) microbiomes. Such a reduction of an individual identity to aspects of its microbiome however is not the only ontological development with significant legal implications prompted by microbiome studies. A rethinking of the definitions of individuals appears also necessary to determine which individuals should be granted new rights. Three distinct options can be considered in law: firstly, a host-associated microbiome, considered as an individual in its own right, may constitute a novel kind of right-holder; secondly, hosts whose interiority transiently emerges from their specific co-construction with some particular microbes may constitute a novel kind of right-holder; thirdly, an entire association of a host and its microbiome, considered the definition of a holobiont, may constitute a novel kind of right holder. Consequently, microbiome studies generally suggest the necessity of introducing a broader ontology within various fields, and to admit an unprecedented spectrum of non-paradigmatic individuals (e.g., new evolutionary individuals, defined by their persistence rather than by their ability to reproduce; transient individuals; and weak individuals [77], to name a few) alongside individuals currently offering more familiarity to most of us.

Together, this ecologization of traditional concepts, this focus on systemic, multi-scalar interactions with microbes, and the adjunction of less familiar individuals into various fields contribute to a fifth strong trend induced by microbiome studies across all disciplines that care about microbiomes: a de-anthropocentrification of our perception of the world. Indeed, microbiome studies prompt us to realize that humans occupy less central or less prestigious positions in nature than we believed we did.

## 4. Towards the Future: Some Outstanding Questions

The above-mentioned epistemic changes only constitute the beginning of a path towards the production of novel knowledge and practices inspired by microbiome studies. As such, they raise major questions, with high transformative potential, some of which are currently under scientific scrutiny.

Strengthening systemic or ecological perspectives:

- Will the original Koch postulate be extended even further than in medical research to demonstrate causal effects of dysbiotic microbial communities, not only on their hosts’ health, but also on the sustainability of their environment, in particular the microbiome impact on elemental biogeochemical cycles and on planetary boundaries?
- Will the extent of the regulation of host gene expression by microbiomes be sufficiently well deciphered to give rise to a ‘microbiomo-genetics’, a genetics of collectives inclusive of both host and microbiome genetic interactions?

Developing a richer ontology:

- Is clade selection prevalent in the microbial world? Whereas better dispersal abilities, or better cellular rejuvenation abilities are expected to favor microbial lineages over others by mere persistence, the ability to engage in productive interspecific interaction (e.g., to laterally exchange genes) constitutes another way through which microbial lineages may enhance their fitness. Therefore, might the persistence of microbiomes also rely on an expanded kind of clade selection, involving members of multiple phylogenetic groups, a form of ‘symbiosis selection’?
- Is it possible to ‘Darwinize microbiomes’, in the same way Gaia was Darwinized, and to identify sets of interacting microbes selected for their collective persistence? Consistently, is the stability of microbial community structure a necessary condition to convey heritable variations?
- Will we be able to delineate and count microbiomes (within an environment, within a host, etc.)? Notably, many biological explanations depend on the ability to identify and count individual members of a given population. For this reason, the terms “individual”, “member of a population”, and “organism” are often used interchangeably. This approach assumes (often wrongly) that there is some sort of homogeneity within and between individuals. It is assumed that biological individuals are constituted of parts that share a common genetic and developmental history and it is often taken for granted that populations are necessarily constituted of these related homogeneous individuals. For most metazoans this assumption may not be a major concern. However, microbiome research highlights that natural entities (individuals or collections of individuals) are often constituted of unrelated heterogeneous individuals functioning as wholes. It is an ever-more accepted fact that unrelated micro-organisms develop complex functioning ecologies with emergent properties or adaptations. This raises fundamental questions about how to identify these collectives, recognize their emergent wholeness, and deal with the fact that they may not always display the fixed boundaries of larger single genome biological individuals. The absence of determinate and fixed boundaries must not deter us from explaining their functional reality and emergent wholeness. The transiency of the boundaries of these systems does not alter the fact that they exist as genuine emergent individual and that we can count them and describe their properties. This being considered, one may wonder how many different microbiomes exist out there, transiently or more permanently?

Moving away from anthropocentrism:

- Will we be able to determine which human traits (if any) escape microbiome influence?
- In order to explain any destabilized biological phenomenon (e.g., to explain host health issues, tipping ecosystems or altered biogeochemical cycles), is it a priori sensible or a microbiome-centric bias to consider changes in microbiome communities, rather than macroscopic biological influences, as critical?
- Will microbiome-based signatures on crime scenes prove to be as effective proxies for non-human species as for human species?

Learning more about these issues can profoundly change our leading philosophical, evolutionary, and legal conceptions of nature.

## 5. Conclusions

Progresses in environmental genomics have deeply transformed our understanding of the living world. While this change is uncontroversial when considering the wealth of data that is now available to describe a microbial community, a profound set of convergent epistemic changes is also ongoing, with consequences affecting the concepts of numerous theories (in evolutionary biology, in ecology, in law, and in philosophy) and associated practices (forensic analyses, medicine, agriculture, and the arts). Therefore, our interdisciplinary work foresees common conceptual enhancements across many fields, driven by microbiome studies, and pushing them towards more ecologized, systemic, interaction-minded, and less anthropocentric approaches.

## Figures and Tables

**Table 1:** Some recognized effects of microbiomes on human health and Earth sustainability.

Human Pathologies Affected by Microbiomes	Planetary Effects of Microbial Communities
Crohn’s disease, Ulcerative colitis, Obesity, Diabetes, Colon cancer, Non Alcoholic Liver Disease, Alcoholic Hepatitis, Atherosclerosis, Hypercholesterolemia, Depression	First evolution of photosynthesis, associated with the Great Oxidation Event, Contribution to the Sulfur cycle, associated with the Permo-Trias crisis, DMS production and global impact on climate

**Table 2:** Some recognized non-human right holders.

Entity	Law
“Nature” as a whole, granting rights to Pacha Mama	Ecuadorian constitution, chapter 7 (2008), Bolivian law (n° 071, 21 December 2010, explicitly including interacting microorganisms)
The Amazonian Forest	Bolivia Supreme Court 5 April 2018)
The Ganges and the Yamuna rivers, and the Whanganui river	Indian law (2017), and New-Zealand law (20 March 2017)
The Gantori and Yamunmonotri glaciers	The Gantori and Yamunmonotri glaciers: (High court of Uttarakhand, Nainital, 30 March 2017)
The ape Cecilia	Mendoza court, 3 November 2016 (n° XPTE.NRO.P-72.254/15)

## Data Availability

Not applicable.
